# Strain improvement and optimization studies for enhanced production of erythromycin in bagasse based medium using *Saccharopolyspora erythraea* MTCC 1103

**DOI:** 10.1007/s13205-013-0186-5

**Published:** 2013-12-18

**Authors:** C. Subathra Devi, Anuj Saini, Shubham Rastogi, S. Jemimah Naine, V. Mohanasrinivasan

**Affiliations:** Industrial Biotechnology Division, School of Biosciences and Technology, VIT University, Vellore, 632014 Tamil Nadu India

**Keywords:** Erythromycin, *Saccharopolyspora erythraea*, Bagasse, UV-mutagenesis

## Abstract

In the present study, *Saccharopolyspora erythraea* MTCC 1103 was used for the enhanced production of erythromycin. To enhance the yield of erythromycin, effects of various parameters such as bagasse concentration, organic nitrogen source, inorganic nitrogen source, pH and temperature were analysed. It was found that bagasse can be used as an alternate carbon source in erythromycin production medium. Erythromycin production in the new formulation of bagasse based medium was found to be 512 mg/L which was 28 % higher than glucose based medium. Strain improvement was done by random UV-mutagenesis. When compared to wild type strain, mutant strain showed 40 % higher yield in production medium. Erythromycin potency assay and HPLC analysis were performed to confirm the presence of erythromycin in the partially purified samples. These optimized conditions could be used for the commercial production of this unique antibiotic which gave significant industrial perspectives.

## Introduction

The discovery of novel antimicrobial compounds is currently the thirst area of research (Jemimah Naine et al. 2012). The production of complex compounds from technically convenient microorganism is an emerging route to the chemical diversity found in the surrounding environment (Jiang et al. 2013). *Saccharopolyspora erythraea* formerly identified as *Streptomyces erythraeus* is a Gram-positive filamentous bacterium. It is one of the most widely exploited strains for the industrial-scale production of erythromycin A. It is a macrolide antibiotic with broad-spectrum activity against Gram-positive pathogens (Oliynyk et al. 2007). In view of the high clinical importance of erythromycin and its derivatives, extensive efforts have been devoted to increase the erythromycin production by *S. erythraea* which has even been studied as a model system for antibiotic production (McDaniel et al. 2001; Leadlay 1997). Meanwhile, erythromycin productivity has also been enhanced by the optimization of fermentation process (El-Enshasy et al. 2008; Zou et al. 2009). Over the past 50 years, the *S. erythraea* strain improvement has been carried out mainly by multiple rounds of random mutagenesis and selection (Adrio and Demain 2006; Carata et al. 2009). Moreover, a series of derivatives of erythromycin, derived by chemical and biotechnological transformation, have been shown to have anti-parasitic, anti-neoplastic, immunosuppressant, neurotrophic, anti-inflammatory and gastro enteric therapeutic activities (Mironov et al. 2004). Erythromycin for medication purpose is generally prescribed in stearate form which is effective against *Streptococcal* and *Staphylococcal infections* (Ishii et al. 1998). Poultry farms are also dependent on this antibiotic for the treatment of bacterial and *Mycoplasma* infections in cattle. Erythromycin has its subtypes as erythromycin A (Er-A), Er-B, Er-C, Er-D, Er-G, etc., out of these types Er-A is clinically most important and specific. It is used for the synthesis of next generation azithromycin and clarithromycin. Er-B and Er-C are produced as by-products during its synthesis which have serious side effects hence not in use (Zou et al. 2010). Mutation-based enhancement in production has gained tremendous importance over the last decade. UV radiation studies have come to the forefront owing to their drastic increase in the production rate. As the antibiotic is produced by a cluster of genes, involving several operons (polycistronic), hence there are very little chances of adverse affects in this technique. Recently, double point mutation of rpsL and rsmG gene in *S erythraea* has been done which has lead to a dramatic increase in erythromycin production (Tanaka et al. 2009).

The primary aim of this work was to search for a cheap carbon source which is also readily available to suffice the basic need of a microbe without compromising the quality of the final product, erythromycin. The remarkable carbon source bagasse is a cheap and readily available substrate for the high yield of antibiotic. It laid the foundation for the future course of action which was to optimize substrate (bagasse) concentration at standard temperature and pH for maximum erythromycin production. After keeping the concentration of substrate as constant, other parameters like nitrogen source (organic and inorganic), pH, temperature were optimized to enhance the production of erythromycin, as well mainly focused on economic considerations without undermining the quality. The strain improvement studies were then carried out on the same MTCC 1103 strain so as to increase the production rate further, which has its immense value on the commercial scale. All these studies underwent had a sole purpose of reducing the cost of the primary antibiotic, erythromycin which is indispensable for the creation of future generation antibiotics like azithromycin.

## Materials and methods

### Microorganism and inoculum preparation

*Saccharopolyspora erythraea* MTCC 1103 was obtained from the Microbial Type Culture Collection, Chandigarh and maintained in ISP-2 solid medium (Enshasy et al. 2007). pH of the medium was adjusted to 7.2 and kept for sterilization. The organism was then inoculated into the sterile medium. The number of spores was counted by spread plate method. A volume of 1 mL of spore suspension (10^10^ spores/mL) was inoculated into 50 mL of seeding medium and incubated at 28 °C for 96 h on a rotary shaker at 200 rpm. 5 % seed culture was inoculated into 250 mL of fermentation medium and incubated at 28 °C for 11 days on a rotary shaker at 200 rpm (Rostamza et al. 2008). All the experiments were performed in triplicates.

### Production medium and cultivation conditions

Production medium composed of (g/L): glucose-20, corn steep liquor-4; NH_4_ (SO_4_)_2_-3; NaCl-2.5 and CaCO_3_-5 were used (Enshasy et al. 2007). The pH of the medium was adjusted to 7.2 and kept for sterilization. Bagasse was obtained from local shops and washed thoroughly with warm water and sun dried. Dried bagasse was converted into fine powder using a grinder. It was sterilized separately and added to the medium before inoculation.

### Determination of biomass dry weight

After 11 days, the production broth was filtered through a Whatman No. 1 filter paper. The biomass on filter paper was carefully transferred to a sterile petri plate. The filtered broth was then centrifuged at 10,000 rpm for 20 min at 4 °C. The pellet obtained was separated from supernatant and added into the earlier petri plate containing biomass. Total biomass containing plate was kept in hot air oven at 50 °C for drying. After drying, biomass was scraped and carefully measured using an accurate weighing balance.

### Standard curve of erythromycin

Erythromycin concentration was determined using UV–visible spectroscopy (Wankhade et al. 2012). Erythromycin standard was prepared by varying concentration ranging from 0.1 to 0.5 mg/mL. Subsequent amount of erythromycin was weighed and dissolved in 1 mL acetonitrile–water solution (1:1) and equal volume of concentrated sulphuric acid was added. This mixture was kept for incubation at 50 °C for 30 min. After incubation period, absorbance of various concentrations was determined at 480 nm using UV–visible spectroscopy. From the obtained values, a standard curve for erythromycin was obtained which was used for estimating unknown concentration of erythromycin.

### Determination of erythromycin

After 11 days incubation, the production broth was centrifuged at 10,000 rpm for 20 min at 4 °C. Then the supernatant was transferred to sterile conical flask and equal volume of chloroform was added to it. Supernatant and chloroform formed two separate phases. The supernatant chloroform mixture was incubated overnight at room temperature on a shaker at 100 rpm. Chloroform was allowed to evaporate and erythromycin was separated. The concentration of erythromycin was then estimated using UV/Vis spectroscopy and compared to the standard curve of erythromycin.

### HPLC analysis

Qualitative estimation of erythromycin was done by HPLC system equipped with a UV detector at 205 nm in C18 column. Acetonitrile:methanol:0.2 M ammonium acetate:water (45:10:10:35) was used as mobile phase at column temperature of 40 °C. Sample injection volume was 50 μL (Rostamza et al. 2008).

### Erythromycin potency

*Micrococcus luteus* MTCC 4821 was employed for the antibacterial assay of the produced antibiotic (Holder and Boyce 1994). Samples were bioassayed against *Micrococcus luteus* MTCC 4821 using agar well diffusion assay. Crude extract from the broth as well as partially purified samples were used for the assay. Standard erythromycin concentration of 1 mg/mL was used as positive control and distilled water as negative control.

### Erythromycin production and growth rate of *Saccharopolyspora erythraea*

The organisms were transferred from ISP-2 slants to 100 mL ISP-2 broth. The broth was incubated at room temperature on a rotary shaker at 200 rpm. Then 5 % (v/v) inoculum was added into 7 different sterile flasks containing production broth and incubated at room temperature on a rotary shaker at 200 rpm. Every day one flask was used for the estimation of erythromycin titre and biomass dry weight. Data were recorded and the values were plotted to obtain the growth curve of the organism.

### Optimization of process parameters for erythromycin production using bagasse as a carbon source

Important process parameters such as carbon source concentration, organic nitrogen source, inorganic nitrogen source, temperature and pH have been optimized to obtain a new formulation which could ensure maximum erythromycin production. The process of optimization encompassed a total of five different experiments concerning five different parameters. Erythromycin titre and dry weight of biomass are the two important parameters which are considered for optimization.

### Strain improvement studies by random UV-mutagenesis

Spore suspensions of *S. erythraea* MTCC 1103 (10^10^ spores/mL) were exposed to UV-irradiation at a distance of 6 cm for 20, 40, 60, 80, 100, 120,140 and 160 s from the UV Strata linker (wavelength 280 nm) (Khaliq et al. 2009). Experiment was carried out in dark to avoid any possible photoreaction. UV exposed spore suspensions were spread on starch casein medium plates and incubated at room temperature for 7 days.

## Results

### Erythromycin production and growth rate of *Saccharopolyspora erythraea*

**Fig. 1:**
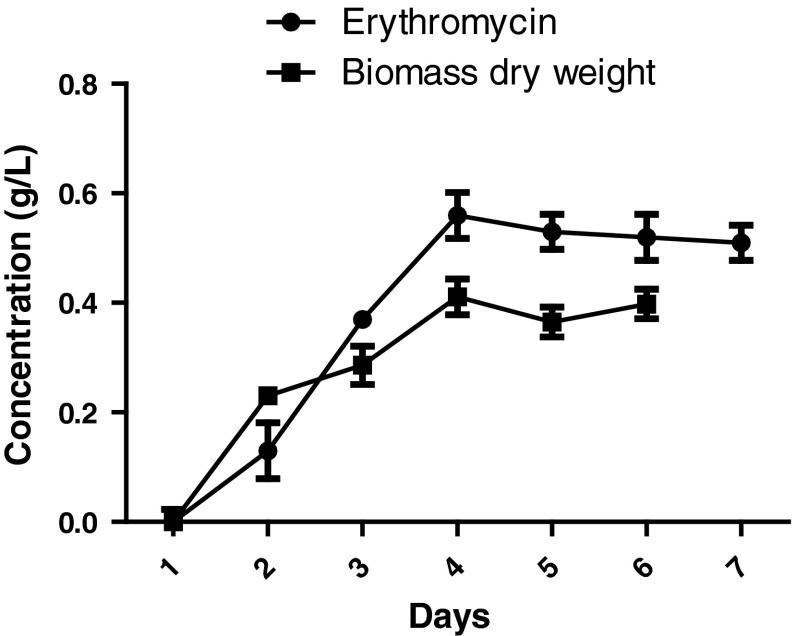
Erythromycin production and growth rate of *Saccharopolyspora erythraea* MTCC 1103 (wild type strain)

**Fig. 2:**
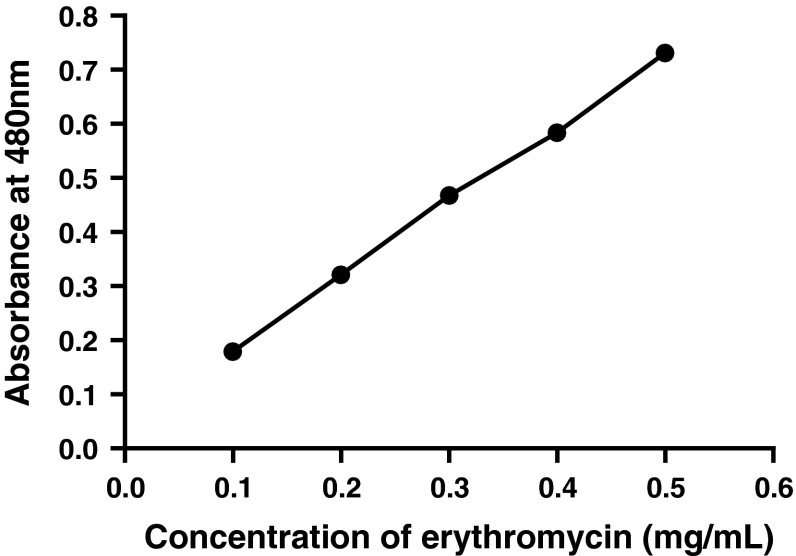
Standard erythromycin curve

Growth rate pattern of the wild type strain was studied in the production medium. Figure 1 shows the kinetics of dry weight biomass and erythromycin yield over a 7 days growth period. Results have suggested that the erythromycin production started from day 3 and it reached maximum on day 5 and remained constant. The maximum erythromycin concentration of 0.411 (g/L) was obtained (Fig. 1). The biomass content increased gradually and it reached the maximum on day 4. After 4 days, it entered the declined phase. The specific growth rate (μ_w_) and doubling time (*t*_dw_) of the wild type strain was found to be 0.735 day^−1^ and 0.94 day, respectively. The standard erythromycin curve was obtained by a linear relationship. It was used for the estimation of unknown concentration of erythromycin in samples (Fig. 2).

## Optimization

### Bagasse concentration

**Fig. 3:**
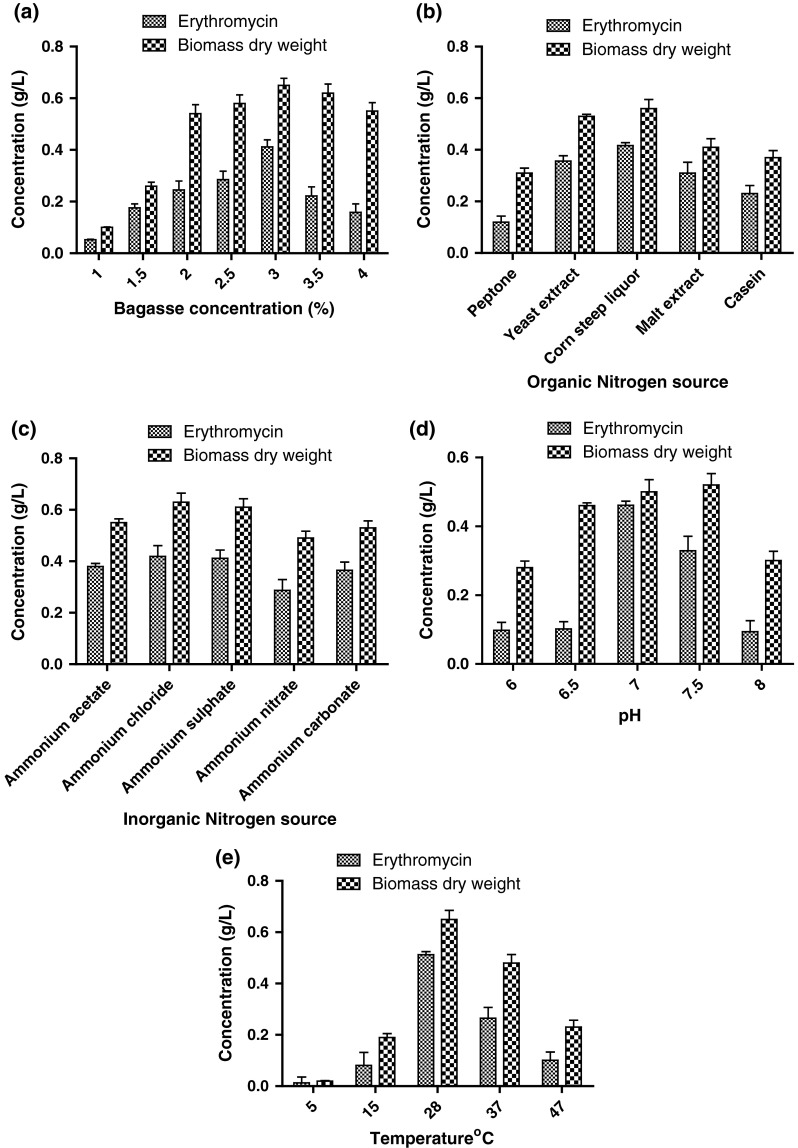
Optimization of **a** bagasse concentration, **b** organic nitrogen source, **c** inorganic nitrogen source, **d** pH, **e** temperature

The effect of bagasse concentration on erythromycin production rate and biomass content has been represented in the Fig. 3a. The maximum concentration of erythromycin (412 mg/L) and biomass content (650 mg/L) was obtained in the production medium supplemented with 3 % bagasse. Minimum production of erythromycin (5.2 mg/mL) and biomass (100 mg/L) was obtained in 1 % bagasse supplemented production medium.

### Organic nitrogen source

Nitrogen source forms a very important component of media for actinomycete growth and antibiotic production. From the results it was found out that corn steep liquor was best followed by yeast extract, malt extract, casein and peptone. The effect of organic nitrogen source on erythromycin production and biomass production has been represented in the Fig. 3b. The other media components were kept constant and 3 % bagasse was used. For corn steep liquor, concentration of erythromycin was found to be 416 mg/L and dry weight of cell biomass as 560 mg/L. In peptone added medium, erythromycin production was as low as 120 mg/L and hence it is least effective for erythromycin production. Yeast extract and malt extract had almost similar erythromycin production rate with 356 and 310 mg/L, respectively.

### Inorganic nitrogen source

Inorganic nitrogen source has been found to be more effective than organic nitrogen source for actinomycetes growth. Various ammonium salts were used for optimization of inorganic nitrogen source. The effect of inorganic nitrogen source on erythromycin production and biomass production has been represented in the Fig. 3c. Corn steep liquor was used as an organic nitrogen source and rest of the parameters were kept constant. Ammonium chloride was found to be best followed by ammonium sulphate. In ammonium chloride supplemented medium, concentration of erythromycin was found to be 419 mg/L and dry weight of cell biomass as 630 mg/L whereas in ammonium sulphate it was found to be 412 and 610 mg/L, respectively. Based on the results, it was concluded that both ammonium chloride and ammonium sulphate can be used effectively as inorganic nitrogen source.

### pH

pH plays an important role in growth of *S. erythraea* and erythromycin production. During the course of this experiment it was found out that the organism is very sensitive to pH. The effect of pH on erythromycin production and biomass production has been represented in the Fig. 3d. It was found that biomass was high at pH 7 and slightly higher at pH 7.5. But the erythromycin production rate was high at pH 7. At pH 7, the concentration of erythromycin was found to be 461 mg/L and dry weight of cell biomass as 500 mg/L. From the results, the optimum pH range for the growth of *S. erythraea* was found to be 7–7.5 whereas the optimum pH for erythromycin production is 7. Thus very high and low pH affected the erythromycin production rate.

### Temperature

Like pH, temperature also plays an important role for the growth of *S. erythraea* and erythromycin production. Actinomycetes are sensitive to temperature. Different range of temperature has been selected ranging from 4 to 47 °C. The effect of temperature on erythromycin production and biomass production has been represented in the Fig. 3e. The optimum temperature for erythromycin production was found to be 28 °C. Highest biomass and erythromycin production was obtained at 28 °C. The betterment of growth and erythromycin production was also observed at 37 °C. Results suggested that optimum temperature for erythromycin production ranges from 25 to 35 °C. At 28 °C, the concentration of erythromycin was found to be 512 mg/L and dry weight of cell biomass as 650 mg/L. These values are obtained after optimizing all the other parameters and thus they indicate maximum erythromycin production and cell biomass dry weight which could be obtained in bagasse based medium.

### Strain improvement studies by UV-mutagenesis

When the *S. erythraea* spore suspension was exposed to UV for less than a minute (10, 20, 30, 40, 50 s), many colonies are observed on the plates. This indicates the organism was not mutated and has grown luxuriously. When the culture was exposed for 1 min, very few colonies were observed on the plate which indicates possible mutation with the strain. No colonies were observed on plates, when the exposure time was more than a minute.

### Erythromycin production and growth rate of mutant *Saccharopolyspora erythraea*

**Fig. 4:**
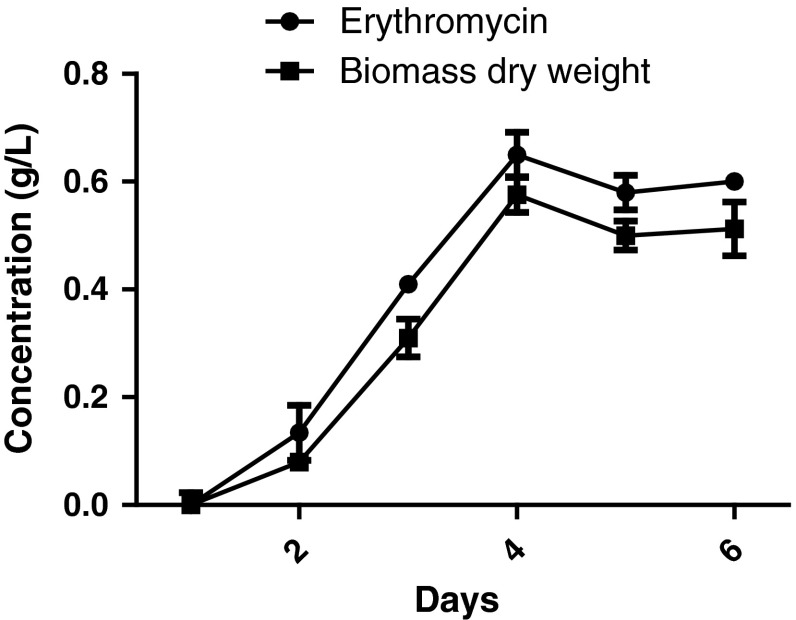
Erythromycin production and growth rate of *Saccharopolyspora erythraea* MTCC 1103 (mutant strain)

The growth rate pattern of the mutant strain was studied in the production broth. When compared to wild type strain, the mutated strain showed maximum growth rate with increased biomass and erythromycin concentration. Specific growth rate (μ_w_) and doubling time (*t*_dw_) of the mutant strain was 0.795 day^−1^ and 0.872 day, respectively. With increased biomass, erythromycin concentration was also found to be higher. Maximum erythromycin production was noticed on day 4 and remained constant. Increase in biomass was obtained from day 2 and reached maximum by day 4. Growth rate of the organisms gradually declined after day 4 (Fig. 4). Erythromycin produced by mutant and wild type strain was found to be 0.576 and 0.411 g/L (Table 1). The results clearly indicate that the productivity of erythromycin was enhanced up to 40 % than the wild strain.

**Table 1:** Comparison of erythromycin production in wild type and mutant strain

Type	Erythromycin (g/L)
Wild type	0.411 ± 0.024
Mutant	0.576 ± 0.012

### Erythromycin potency assay

**Fig. 5:**
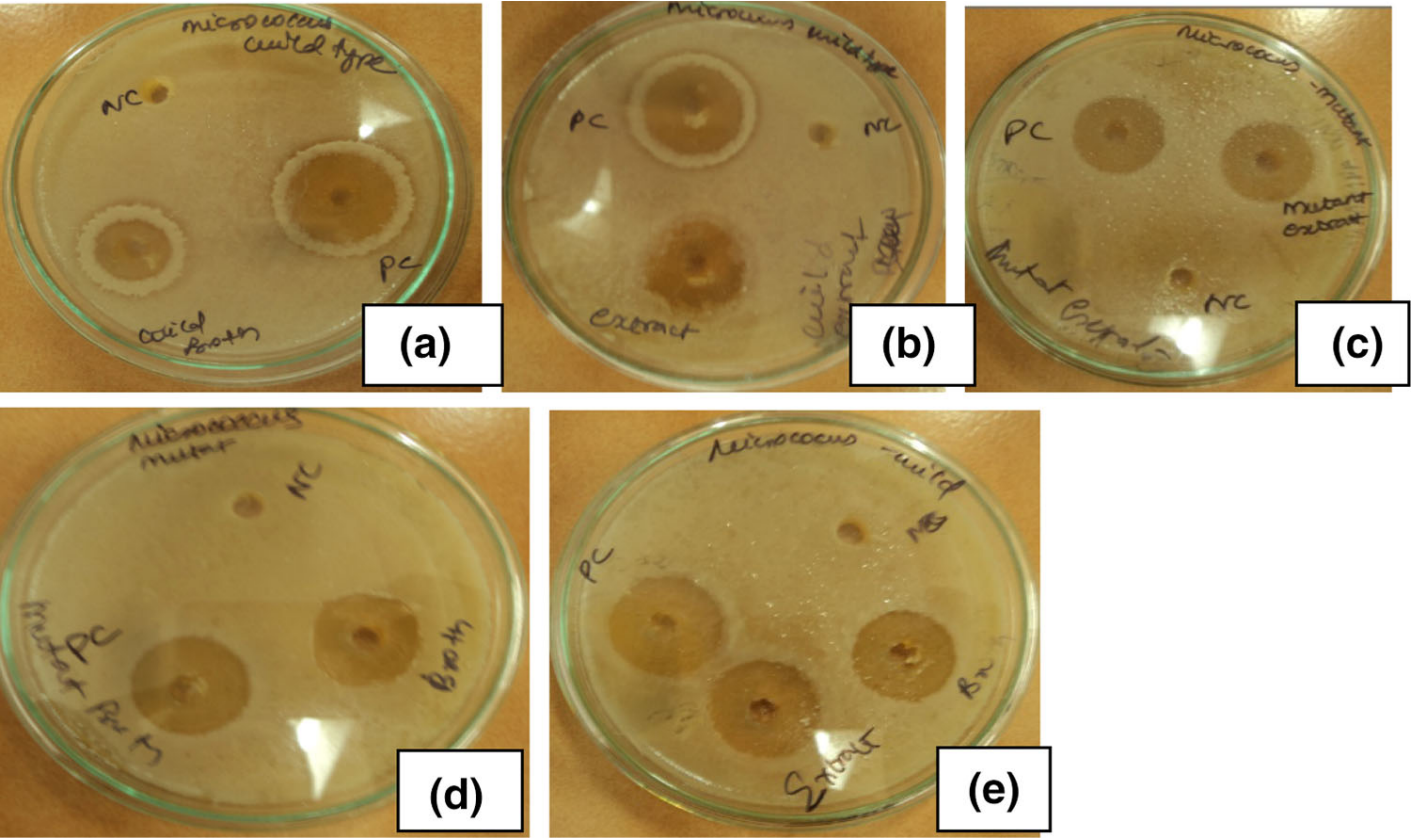
Erythromycin potency against *Micrococcus luteus* MTCC 4821. **a** Crude extract of wild type strain in standard production medium, **b** partially purified extract of wild type strain in standard production medium, **c** crude extract of mutant strain in standard production medium, **d** partially purified extract of mutant strain in standard production medium, **e** partially purified and crude extract of wild type strain in bagasse medium

The clear inhibitory zone of 20 and 24 mm against *Micrococcus luteus* MTCC 4821 was observed for crude erythromycin extracted from wild type and mutated strain. The partially purified extract showed 18 and 22 mm respectively (Fig. 5a–e; Table 2). When compared to crude samples, maximum zone of inhibition was observed in partially purified samples.

**Table 2:** Zone of inhibition of wild type and mutant in standard production medium and wild type in bagasse medium against *Micrococcus luteus* MTCC 4821

	Diameter of zone of inhibition (in cm)		
	Erythromycin produced by wild type strain in production broth	Erythromycin produced by mutant strain in production broth	Erythromycin produced by wild type strain in bagasse production broth
Positive control (standard erythromycin 1 mg/mL)	2.6 ± 0.134	2 ± 0.05	2 ± 0.052
Negative control (distilled water)	0	0	0
Crude extract	2 ± 0.067	1.8 ± 0.034	1.9 ± 0.21
Partially purified extract	2.4 ± 0.05	2.2 ± 0.09	2 ± 0.19

### HPLC analysis

**Fig. 6:**
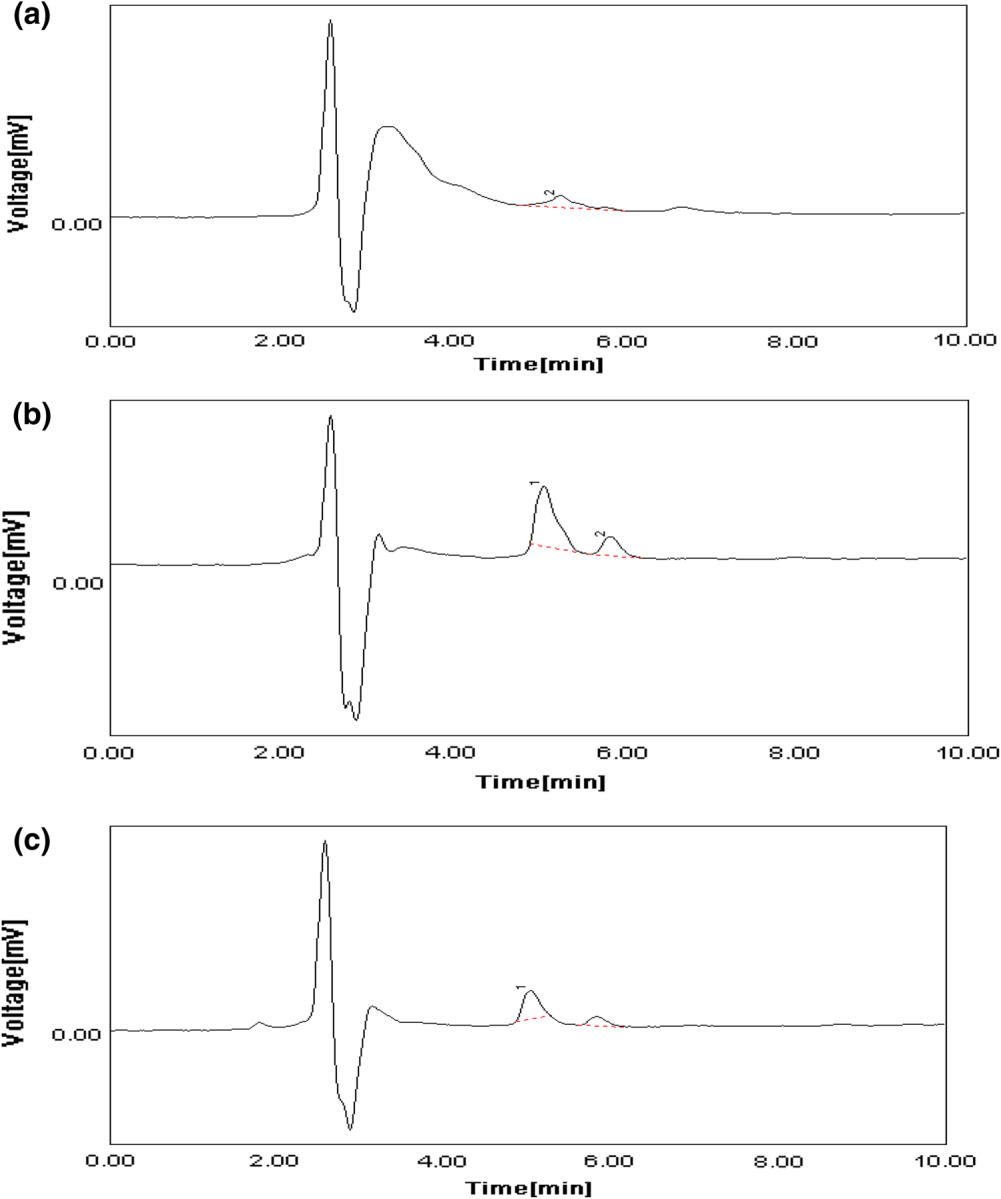
HPLC chromatogram of partially purified samples. **a** Wild type strain in standard medium, **b** mutant strain in standard medium, **c** wild type strain in bagasse medium

Samples extracted from wild type and mutant strain of *S. erythraea* in standard production medium and wild type strain in bagasse medium were subjected to HPLC analysis. Figure 6a–c shows the HPLC profile of erythromycin. Retention time and area represent the amount of erythromycin present in crude extracts (Table 3). Hence the retention time of all the peaks were found closer to 5 min which corresponds to the standard erythromycin retention time (Abdulla et al. 2008).

**Table 3:** Retention time and area under the peak for HPLC chromatogram

Strain and medium type	Retention time (min)	Area under the peak (mV s)
Wild type in standard medium	5.2333	133.4693
Mutant in standard medium	5.0500	289.2130
Wild type in bagasse medium	5.0167	168.2511

## Discussion

The importance of a new and cheap carbon source for erythromycin production was uncovered in the previous studies. Different production media are used in the fermentation industry. Molasses has been widely used in the production medium of erythromycin (Enshasy et al. 2007). In this research, effect of bagasse on growth of *S. erythraea* MTCC 1103 and erythromycin production was evaluated. The results suggested that bagasse can be effectively used for production of erythromycin. Bagasse concentration has been identified as an important parameter which affects erythromycin production as it was being used as a carbon source instead of glucose. This is the foremost report of bagasse being used for erythromycin production. Lower bagasse concentration could have resulted in low yield and higher concentration can result in lower yield because of substrate inhibition. The optimum bagasse concentration was found to be 3 % (w/v) in comparison to 2 % (w/v) conventionally used (Enshasy et al. 2007). Erythromycin production in bagasse based medium was found to be 512 mg/L which was 28 % higher compared to glucose based medium. In molasses-based medium, the antibiotic production reached up to 600 mg/L using *S. erythraea* NCIMB 8594 which is comparable to bagasse based medium. Considering the economy of sources, bagasse was better than glucose. Bagasse is cheaper than glucose and molasses. Higher antibiotic yield was obtained in glucose based medium and comparable yield to molasses-based medium.

Optimization of media parameters is essential to improve secondary metabolites production. Bagasse concentration was one of the most important parameter, as less concentration would mean compromising the production rate and higher concentration of bagasse can lead to substrate inhibition as seen in case of glucose. This lead to transient repression of antibiotic formation, maximum repression was reported at 20 mg/mL glucose concentration (Escalante et al. 1982). The optimum pH and temperature were found to be 7.0 and 28 °C. Corn steep liquor was found to be the best organic nitrogen source which corresponds to earlier findings (Enshasy et al. 2007). When compared to other salts ammonium chloride was found to be the best inorganic nitrogen source for the production of erythromycin. Earlier studies using molasses-based formulation also showed similar results with ammonium sulphate and ammonium chloride (Enshasy et al. 2007). The mutated organism showed higher growth rate and erythromycin production when compared to wild type. Earlier study involving *S. erythraea* FL2267 showed 50 % increase in antibiotic production by engineering the methylmalonyl-CoA metabolite node (Reeves et al. 2007). UV-mutagenesis was much easier to perform than metabolic engineering of specific pathways and results are comparable too.

Several studies were previously reported on *streptomyces* (Sejiny 1991). The growth kinetics of wild type and mutant *S. erythraea* was compared to the specific growth rate constant. i.e. μ_m_ is greater than μ_w_, and doubling time of mutant was less than the wild type strain, i.e. *t*_dm_ is lesser than *t*_dw_. This indicates that mutant strain grew faster than wild type strain. HPLC profile of the samples confirmed the presence of erythromycin. Earlier studies on HPLC chromatogram of erythromycin indicates that retention time of 5.88 min (Abdulla et al. 2008). Area under the peak for wild type strain using bagasse as carbon source is more than that of wild type using glucose as carbon source and less than the mutant strain. This suggests that erythromycin produced by wild type in bagasse is more than that produced in glucose. Also the erythromycin produced by mutant is greater than wild type in glucose and bagasse which is correlated with our earlier findings.

## Conclusion

The primary aim of the study was to find a new and cheap carbon source for erythromycin production and enhance the production of erythromycin by random UV-mutagenesis of the strain *S. erythraea*. After identifying bagasse as a new carbon source it was important to optimize the process parameters for maximum production of erythromycin. Extensive literature review led to the identification of five process parameters i.e. bagasse concentration, organic nitrogen source, inorganic nitrogen source, pH and temperature. It was found out that sucrose can easily be replaced by bagasse for erythromycin production. Bagasse is cheap and easily available as waste from sugarcane industries. By random UV-mutagenesis studies, a mutant strain was obtained which showed 40 % increase in erythromycin production which is significant. The mutant strain grew faster than the wild type strain. Erythromycin is one of the most important antibiotics used in day-to-day basis and is widely manufactured and used. Thus if it is produced at a cheaper rate it would be good for the society. Further research can include production of erythromycin in bagasse medium using mutant strain and optimizing parameters for enhanced production.
